# Killer Peptide: an antibody-derived self-assembling peptide bridging antimicrobial and host-defense mechanisms

**DOI:** 10.1093/femsre/fuag026

**Published:** 2026-06-18

**Authors:** Miguel Fernandez de Ullivarri, Colin O’Sullivan, R Paul Ross, Colin Hill

**Affiliations:** APC Microbiome Ireland, University College Cork, Cork, T12YT20, Ireland; School of Microbiology, University College Cork, Cork, T12Y337, Ireland; APC Microbiome Ireland, University College Cork, Cork, T12YT20, Ireland; School of Microbiology, University College Cork, Cork, T12Y337, Ireland; APC Microbiome Ireland, University College Cork, Cork, T12YT20, Ireland; School of Microbiology, University College Cork, Cork, T12Y337, Ireland; APC Microbiome Ireland, University College Cork, Cork, T12YT20, Ireland; School of Microbiology, University College Cork, Cork, T12Y337, Ireland

**Keywords:** β-glucan recognition, peptide self-assembly, multi-kingdom activity, programmed cell death, immunomodulation, anti-idiotypic antibodies

## Abstract

Antimicrobial resistance and persistent biofilm-associated infections continue to drive the search for peptide-based anti-infective agents with mechanisms distinct from conventional antibiotics. Killer Peptide (KP) is an antibody-derived decapeptide originally identified through yeast killer toxin mimicry studies and has emerged as a distinctive example of a multifunctional bioactive peptide. Experimental studies have reported particularly strong antifungal activity, together with activity against selected bacteria, biofilms, viruses, and protozoa, as well as immunomodulatory effects in preclinical models. Mechanistically, KP appears to act through a multistep process involving target-surface recognition, cellular internalization, induction of intracellular stress pathways, and reversible self-assembly into fibrillar structures that may support localized peptide retention. In parallel, KP has been reported to influence innate and adaptive immune responses, suggesting potential host-defense-enhancing properties. In this review, we critically reassess two decades of KP research, covering its molecular origin, structural features, antimicrobial spectrum, mechanisms of action, engineered derivatives, and translational prospects. We also examine current limitations, including the relatively narrow evidence base, incomplete pharmacological characterization, and the need for independent validation across disease models. KP represents a useful conceptual framework for the development of next-generation multifunctional peptides integrating antimicrobial, pathogen-triggered assembly, and immunomodulatory properties.

## Teaser

Killer Peptide (KP) is an antibody-derived peptide whose self-assembly, antimicrobial activity, and immunomodulatory effects make it a useful model for multifunctional anti-infective design.

## Introduction

The global rise in antimicrobial resistance (AMR) is accelerating the search for novel agents with broad anti-infective activity and potentially reduced propensity for resistance development. Peptide-based therapeutics, particularly those derived from innate or immunological pathways, offer a promising strategy. Among these, the Killer Peptide (KP, AKVTMTCSAS) is a notable example of an antibody-derived antimicrobial agent with both microbicidal and immunomodulatory properties (Polonelli et al. 2003, Magliani et al. 2008). Since its initial characterization in the early 2000s, KP has demonstrated robust antifungal activity and more limited but emerging activity against selected bacterial, viral, and protozoal systems, while exhibiting low toxicity toward mammalian cells in preclinical models (Magliani et al. 2011, Ferrari et al. 2016, 2020, Sala et al. 2019, 2024).

Compared to conventional antimicrobial peptides (AMPs), current evidence suggests that KP binds β-1,3-glucan-containing microbial surfaces, undergoes cellular uptake, and induces stress-associated programmed cell death pathways (Savoia et al. 2006, Pertinhez et al. 2009, Magliani et al. 2011, Paulone et al. 2017, Giovati et al. 2018). Moreover, KP can self-assemble into fibrillar supramolecular assemblies upon interaction with β-1,3-glucans, which may enhance local retention and sustained activity (Pertinhez et al. 2009). In parallel, KP exerts immunomodulatory effects by activating antigen-presenting cells and promoting the secretion of Th1-type cytokines (Cenci et al. 2006, Ferrari et al. 2016, 2020). These combined actions position KP as a multifunctional antimicrobial and immunomodulatory peptide.

Although the KP literature remains relatively limited in size compared with more established antimicrobial peptide fields, important developments have emerged since the previous dedicated review by Magliani et al. (2011). These include mechanistic studies on peptide self-assembly, expanded investigation of immunomodulatory effects, rational engineering of KP analogues, activity against resistant biofilms and ESBL-producing bacteria, and early exploration of formulation strategies. Moreover, many of these findings are distributed across microbiology, immunology, peptide engineering, and supramolecular assembly literature and have not previously been integrated into a unified critical perspective. Accordingly, this review summarizes studies published since 2011 and re-evaluates KP in the context of antimicrobial activity, self-assembly, and host-directed effects.

## Positioning KP within established AMP mechanistic classes

Antimicrobial peptides encompass mechanistically diverse molecules that kill pathogens through membrane disruption, intracellular targeting, immune modulation, or combinations thereof. Comparison with established AMP classes provides a useful framework for understanding KP activity.

Classical membrane-active peptides such as melittin, magainins, and nisin act primarily through rapid disruption of membrane integrity. Melittin and magainins form pores or destabilize lipid bilayers through amphipathic helix formation (Memariani et al. 2020, Di Somma et al. 2022), whereas nisin binds lipid II and forms pores in susceptible Gram-positive bacteria (Kramer et al. 2004, Panina et al. 2021, Field et al. 2023).

By contrast, intracellularly active peptides such as buforin II penetrate cells with limited lysis and target nucleic acids or other internal components, thereby suppressing essential cellular functions (Park et al. 1998, Kobayashi et al. 2004, Hao et al. 2013). Similar intracellular targeting has been described for indolicidin and related peptides (Cardoso et al. 2019). These peptides can translocate across membranes without extensive lysis, either through transient membrane perturbation or other non-lytic translocation mechanisms (Shah et al. 2016).

A third functional group includes host-defense peptides such as LL-37 and human β-defensins, which combine antimicrobial activity with immunomodulatory functions including cytokine induction, chemotaxis, and regulation of inflammatory responses (Pahar et al. 2020, Guryanova and Ovchinnikova 2022).

Although KP is not a classical innate AMP, its antimicrobial and immunomodulatory properties justify comparison with established AMP classes. KP shares features with several AMP groups rather than corresponding closely to a single mechanistic category. Current evidence suggests that KP activity involves target-surface recognition, intracellular stress induction, reversible self-assembly, and modulation of host immune responses, although the mechanisms governing membrane interaction and cellular entry remain incompletely understood. These properties distinguish KP from many canonical AMPs and may contribute to its reported broad-spectrum potential, although evidence remains strongest for fungal systems.

## Structure and origin

**Figure 1:**
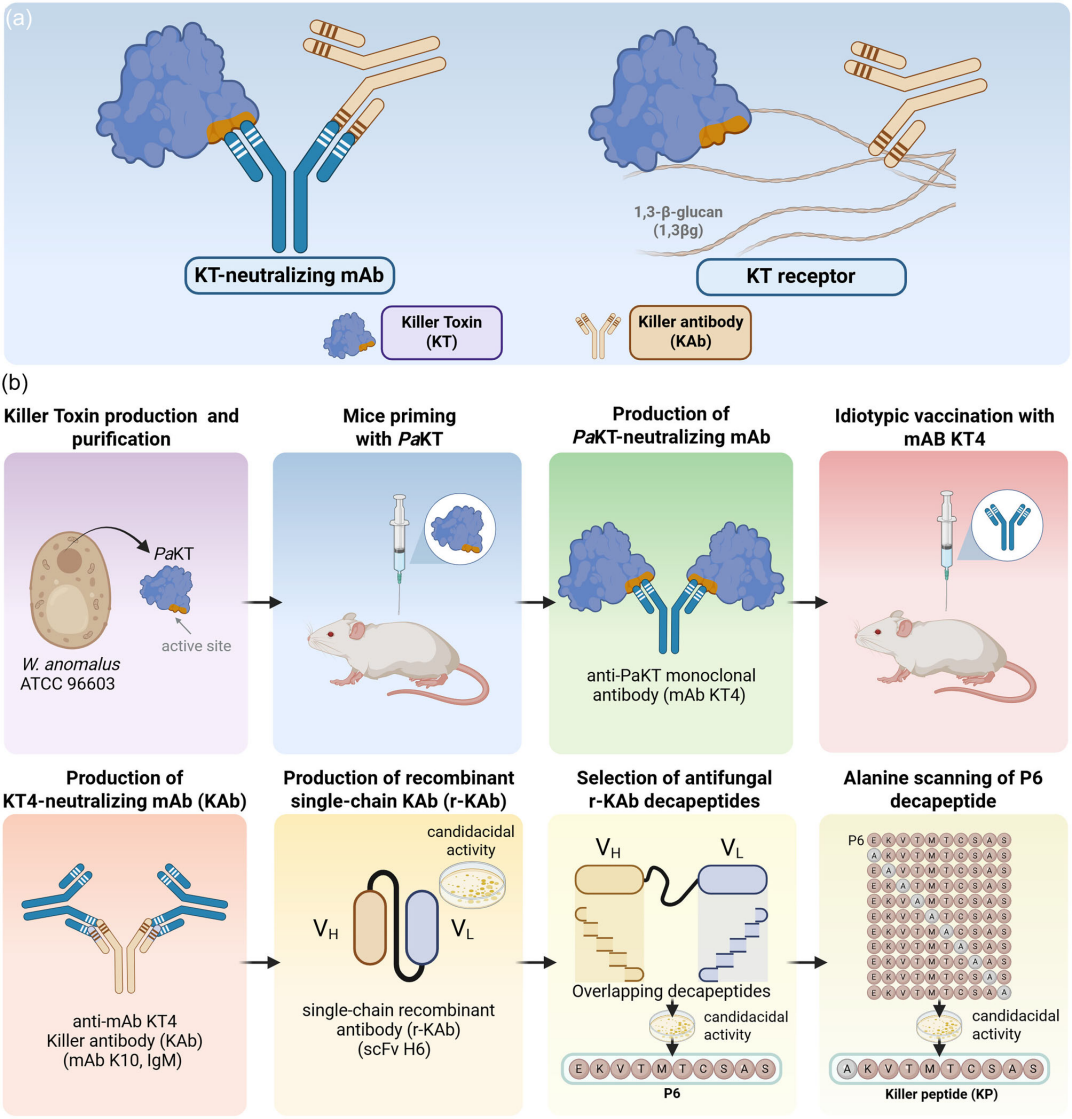
Historical origin and molecular derivation of Killer Peptide (KP). (a) Representation of the killer antibody (KAb) as an immunological mimotope of a killer toxin (KT) by their binding capacity to the KT-neutralizing antibody and the KT receptor; (b) schematic overview of the experimental pathway leading to KP discovery. Studies on yeast killer toxins enabled generation of anti-idiotypic antibodies mimicking killer toxin epitopes, from which recombinant antibody fragments were developed. Sequence analysis and peptide mapping ultimately led to the identification of the decapeptide KP (AKVTMTCSAS).

KP is a decameric peptide derived from the light-chain variable region CDR sequence of a recombinant antibody. This antibody was raised as an anti-idiotype to a yeast killer toxin (KT) and mimics the receptor-binding properties of the toxin active site (Fig. 1). KP is a minimal, antibody-derived mimic of the original yeast KT. The presence of a cysteine residue enables intermolecular disulfide-linked dimerization, which is required for fungicidal activity (Magliani et al. 2011).

Some yeast strains, termed “killer yeasts”, secrete killer toxins (KTs), antimicrobial proteins or glycoproteins that eliminate susceptible micro-organisms via receptor-mediated mechanisms. Among them, the KT produced by *Wickerhamomyces anomalus* ATCC 96603 (formerly *Pichia anomala* ATCC 96603), PaKT, exhibits broad antifungal spectrum by targeting β-glucan-containing cell wall structures in pathogens such as *Candida albicans*. However, PaKT’s toxicity, immunogenicity, and instability under physiological conditions make it unsuitable for therapeutic use (Magliani et al. 2008).

Researchers used PaKT as a template to generate functional mimics that circumvent these limitations. Initially, a monoclonal antibody (mAb KT4) capable of neutralizing PaKT was produced. The authors subsequently used this antibody to generate anti-idiotypic antibodies, termed “killer antibodies” (KAbs), which retained PaKT’s microbicidal activity by mimicking its interaction with β-glucan receptors (Magliani et al. 2008).

Recombinant forms of KAbs enabled further size and complexity reduction. The authors then synthesized overlapping decapeptides from the variable regions of a single-chain recombinant KAb (scFv H6) and identified a candidate peptide named P6. Alanine scanning of P6 generated the peptide KP, which showed enhanced candidacidal activity. Competition assays with KAbs and laminarin (a soluble β-1,3-glucan) showed that KP preserved the receptor-binding specificity of its parental r-KAb (Magliani et al. 2008). These developmental steps are illustrated in Fig. 1.

KP thus represents a minimal functional unit developed from PaKT through an anti-idiotypic pathway. This development demonstrates an alternative route to peptide discovery—distinct from genome mining or host-defense libraries—that may help identify functionally optimized, non-toxic antimicrobial candidates.

### Physicochemical properties

**Figure 2:**
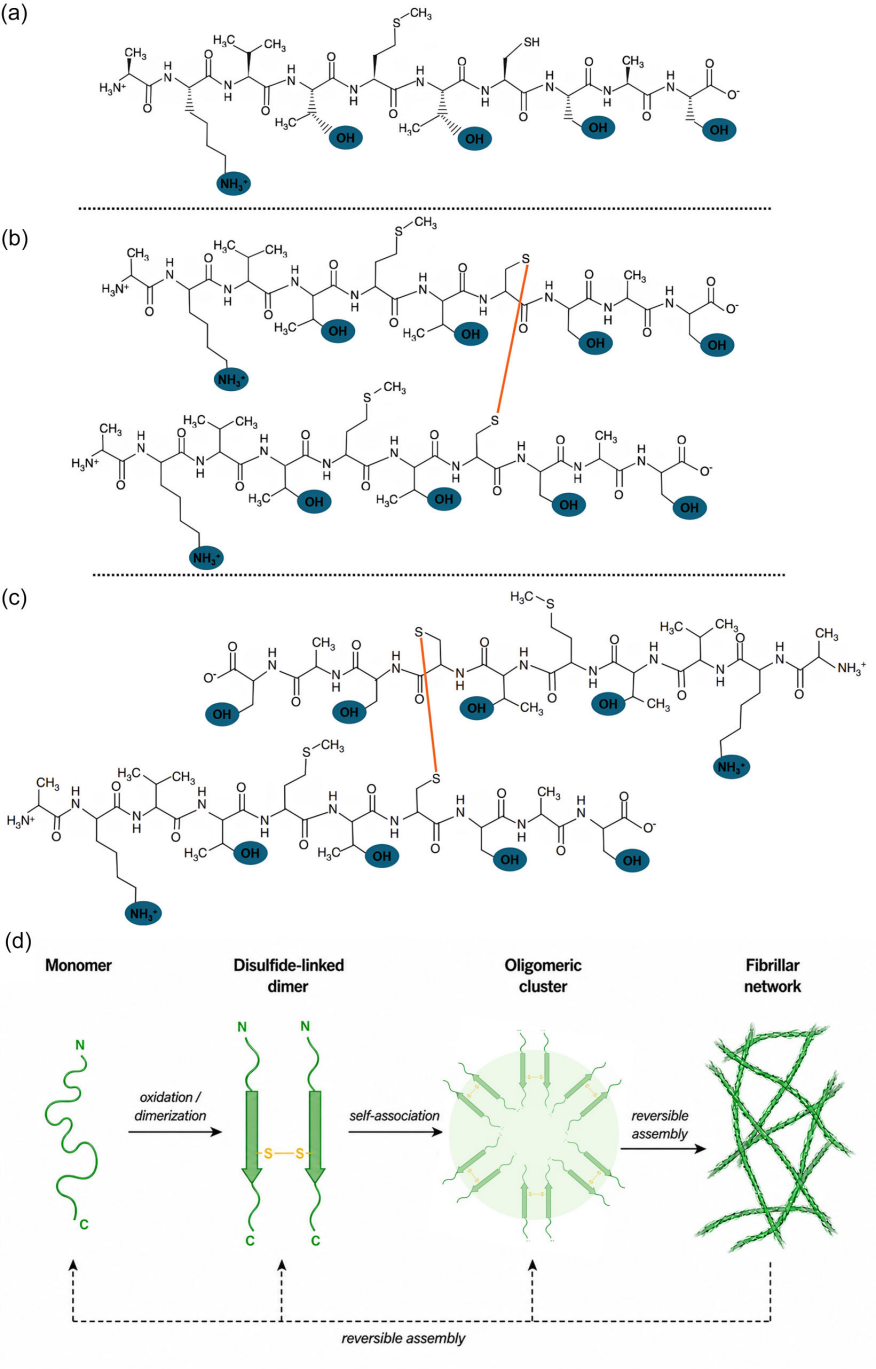
Proposed molecular conformations of KP. (a) monomer; (b) parallel disulfide-linked dimer; and (c) antiparallel dimer. Disulfide bond (orange) and polar residues (blue) on the hydrophilic face, which may participate in hydrogen bonding networks, are indicated. Adapted from Magliani et al. (2011). (d) Conceptual hierarchical assembly pathway of KP from monomeric peptide to disulfide-linked dimers and higher-order fibrillar assemblies. Structural intermediates are schematic and not intended as experimentally resolved conformations.

KP is a 10-residue peptide (AKVTMTCSAS) with a net positive charge (+1) at physiological pH conferred by a single lysine residue. It contains both polar and hydrophobic amino acids but lacks the amphipathic α-helical structure characteristic of many canonical AMPs (Fig. 2). Structural studies instead suggest that KP adopts predominantly β-strand or disordered conformations and can self-associate into supramolecular aggregates. Intermolecular disulfide bond formation between cysteine residues mediates peptide dimerization (Ciociola et al. 2021b, Polonelli et al. 2003, Pertinhez et al. 2009). KP can be synthesized chemically or expressed recombinantly; e.g. display on plant virus particles has been explored to enhance antimicrobial activity (Donini et al. 2005).

### Discovery and initial studies

KP was first validated in antifungal models. Polonelli et al. demonstrated that KP kills *C. albicans* and *Cryptococcus neoformans in vitro* and exerted therapeutic efficacy *in vivo*. KP treatment reduced fungal burden in multiple organs in a murine model of systemic *Paracoccidioides brasiliensis* infection (Polonelli et al. 2003, Cenci et al. 2004, Travassos et al. 2004). The authors used a scrambled-sequence peptide containing identical amino acid composition but randomized sequence order as a negative control, which showed no activity *in vitro* or *in vivo*, confirming KP’s sequence-specific antifungal effect. KP was well tolerated in treated mice and rats, with no signs of toxicity or weight loss (Polonelli et al. 2003, Cenci et al. 2004, Travassos et al. 2004). These early studies demonstrated potent antifungal activity and a favourable preliminary safety profile, justifying further exploration of KP as an antimicrobial candidate (Magliani et al. 2008).

## Spectrum of activity

KP has been reported to display antimicrobial activity spanning multiple pathogen groups, including fungi, selected bacteria, viruses, and protozoa. However, the depth and quality of evidence vary substantially among target classes, with fungal pathogens representing by far the most extensively studied and experimentally supported indication.

**Figure 3:**
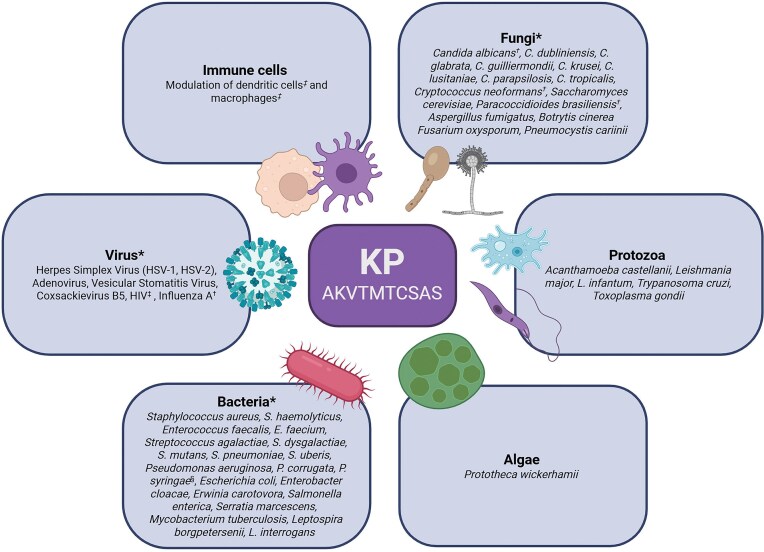
Overview of KP’s antimicrobial spectrum across major pathogen classes and immunomodulatory activity. *Including drug-resistant strains; *^†^In vivo; ^‡^Ex vivo; ^§^In planta*. Redrawn and updated from Magliani et al. (2011).

The antimicrobial spectrum across pathogen groups is illustrated in Fig. 3, while Table 1 summarizes representative potency values reported across systems. These studies demonstrate substantial variability in effective concentrations between pathogen classes, However, direct comparison of potency values between pathogen classes should be interpreted cautiously because assay conditions and experimental endpoints vary substantially among studies. Antifungal activity is generally observed at low micromolar concentrations, whereas antibacterial, antiviral, and antiparasitic effects often require higher concentrations or show greater variability depending on the experimental model.

**Table 1:** Reported antimicrobial and anti-infective activity of Killer Peptide (KP) across biological targets.

Target Class	Target species/organism	Assay type	Variable^	Representative potency (μM)*	Experimental context	Reference
Yeast	*Candida albicans*	Dose-response killing assay (CFU count)	EC_50_	0.31–0.67	Planktonic cells in H_2_O; 6 h incubation	Ciociola et al. 2021b, Paulone et al. 2017
Yeast	*C. albicans* SC5314	Biofilm biomass reduction (Crystal Violet)	EC_50_	137.55	Mature 48h-old biofilm grown on polystyrene plates; 6 h incubation	Paulone et al. 2017
Yeast	*Cryptococcus neoformans*	Dose-response killing assay (CFU count)	EC_50_	10–20	Capsular and acapsular strains (yeast) in RPMI; 48 h incubation	Cenci et al. 2004
Mold	*Botrytis cinerea*	Minimal inhibitory concentration	MIC	120	MIC required to prevent complete spore germination to hyphae; 6 h incubation	Donini et al. 2005
Mold	*Fusarium oxysporum*	Minimal inhibitory concentration	MIC	100	MIC required to prevent complete spore germination to hyphae; 6 h incubation	Donini et al. 2005
Bacteria	*P. syringae*	Dose-response killing assay (CFU count)	EC_50_	32–44	Cells in PBS; 6 h incubation	Donini et al. 2005
Bacteria	*Erwinia carotovora*	Dose-response killing assay (CFU count)	EC_50_	50	Cells in PBS; 6 h incubation	Donini et al. 2005
Bacteria	*Escherichia coli* ATCC 25 922	Dose-response killing assay (CFU count)	EC_50_	0.309	Cephalosporyn-sensitive *E. coli*; Cells in H_2_O; 5 h incubation	Artesani et al. 2024
Bacteria	ESBL-producing *E. coli*	Dose-response killing assay (CFU count)	EC_50_	0.751–1.099	Cephalosporyn-resistant *E. coli* strains; Cells in H_2_O; 5 h incubation	Artesani et al. 2024
Bacteria	*Enterococcus faecalis* ATCC 29 212	Dose-response killing assay (CFU count)	EC_50_	4.52	Cells in H_2_O; 5 h incubation	Mergoni et al. 2020
Bacteria	*E. faecalis* ATCC 29 212	Biofilm viability reduction (Alamar blue)	EC_50_	123.3	Early biofilm formation on polystyrene plates; Alamar Blue assay	Mergoni et al. 2021
Virus	Influenza A virus H7N1	Dose-response antiviral replication assay	EC_50_	2.7	*In vitro*, Influenza A-infected epithelial cell monolayers; PFU-based viral yield; post-infection treatment	Conti et al. 2008
Virus	Herpes simplex (HSV)	Dose-response antiviral assay	IC_50_	13.6 (HSV-1); 2.6 (HSV-2)	Infection models in Vero cells; PFU-based viral yield	Sala et al. 2024
Virus	HIV-1	Antiviral replication assay	EC_50_	0.1–0.5^#^	Infection models in PBMCs; p24 ELISA/viral RNA; non dose-response	Casoli et al. 2006
Protozoa	*Leishmania spp*.	Dose-response killing assay	EC_50_	58 (*L. major*); 72 (*L. infantum*)	*In vitro*, extracellular *Leishmania* promastigotes in culture medium; 24 h treatment	Savoia et al. 2006
Protozoa	*Toxoplasma gondii*	Dose-response killing assay	EC_50_	140^#^	*In vitro*, extracellular *T. gondii* tachyzoites (pre-infection model in HFF system); 2 h incubation	Bucella et al. 2025
Protozoa	*Acanthamoeba* spp.	Dose- and time-response killing assay	EC_50_	10–25^#^	*In vitro*, extracellular *A. castellanii* trophozoites in PYG medium; 0–3 day incubation	Fiori et al. 2006

^ EC50: Concentration required to achieve 50% of the maximal observed effect; IC50: Concentration required to inhibit a biological process or activity by 50%, MIC: The lowest concentration that prevents visible growth of a micro-organism after incubation.

* Values originally reported in µg/ml were converted to approximate µM values assuming a molecular weight of ∼1 kDa for KP; therefore, 1 µg/ml was considered approximately equivalent to 1 µM.

# Values not explicitly reported in the original studies were estimated from the published experimental data or figures.

### Antifungal activity

Antifungal activity remains the most robustly characterized component of the KP literature. Early studies demonstrated therapeutic efficacy of KP or related engineered antibody-derived peptides in experimental mucosal and systemic candidiasis, establishing proof-of-concept for *in vivo* antifungal application (Polonelli et al. 2003). Subsequent work confirmed activity against *C. albicans* and multiple non-*albicans Candida* species, including strains with reduced susceptibility to conventional azoles (Manfredi et al. 2005). Mechanistic studies further showed that KP can induce apoptosis-like death pathways in *C. albicans*, supporting a regulated intracellular mode of killing rather than simple lytic toxicity (Ciociola et al. 2021b, Paulone et al. 2017). As summarized in Table 1, KP antifungal activity is consistently observed at low micromolar concentrations in planktonic assays, with EC_50_ values typically below 1 µM for *C. albicans*, supporting fungi as the most potent and reproducible target class.

Beyond candidiasis, KP has shown activity against other medically relevant fungi. Anticryptococcal effects were reported against *C. neoformans* (Cenci et al. 2004), while therapeutic benefit was demonstrated in murine paracoccidioidomycosis models caused by *P. brasiliensis* (Travassos et al. 2004). In veterinary mycology, KP also had *in vitro* and *in vivo* efficacy against *Malassezia pachydermatis* in canine otitis externa (Cafarchia et al. 2014). Collectively, these findings indicate that fungal pathogens remain the clearest translational focus for KP development.

An additional strength of KP is activity against fungal biofilms. KP impaired *C. albicans* biofilm formation and reduced mature biofilm biomass *in vitro*, an important property given the role of biofilms in chronic mucosal and device-associated infections (Paulone et al. 2017). Because biofilm tolerance often limits conventional antifungal therapy, this feature may be particularly relevant for future topical or adjunctive applications.

### Antibacterial activity

Compared with fungi, antibacterial evidence is more limited and generally more recent. Native KP appears less potent than many classical antibacterial peptides. As summarized in Table 1, antibacterial activity is observed over a broader and less consistent concentration range than for fungal targets, with reported EC_50_ values varying substantially across strains and assay conditions. In particular, KP is effective against both Gram-positive bacteria such as *E. faecalis* and *Staphylococcus aureus* (Donini et al. 2005, Mergoni et al. 2020, 2021) and Gram-negative bacteria such as *Pseudomonas syringae, Erwinia carotovora* (tested as models of phytopathogens), and cephalosporin-resistant ESBL-producing *Escherichia coli* strains (Donini et al. 2005, Artesani et al. 2024).

### Antiviral activity

KP has also been reported to display antiviral activity against several enveloped and non-enveloped viruses. Initial *in vitro* studies demonstrated particularly strong activity against Herpes Simplex Virus (HSV), while more limited or partial inhibitory effects were observed against Vesicular Stomatitis Virus (VSV), Adenovirus, and Coxsackievirus B5 depending on the peptide variant and assay conditions (Sala et al. 2019). More recently, treatment with KP reduced infectious titres of HSV-1 by approximately 3.5 log and HSV-2 by approximately 4.1 log in cell culture (Sala et al. 2024). Mechanistic assays indicated that KP directly interacts with HSV virions, impairing virus adsorption and entry into host cells. KP also retained activity against acyclovir-resistant HSV strains and exhibited synergistic effects when combined with acyclovir. Earlier studies further reported inhibition of HIV-1 and Influenza A virus replication, including therapeutic activity in murine influenza models, suggesting that KP may interfere with viral infection through mechanisms distinct from those of conventional antivirals (Casoli et al. 2006, Conti et al. 2008a).

### Antiparasitic and protozoal activity

KP has additionally shown activity against several eukaryotic parasites and free-living protozoa. Growth inhibition and ultrastructural damage were reported in *Leishmania major* and *L. infantum* promastigotes (Scutera et al. 2005, Savoia et al. 2006). Activity against *Toxoplasma gondii* tachyzoites has also been described (Giovati et al. 2018), extending the spectrum to apicomplexan parasites. Earlier studies further reported acanthamoebicidal activity against *Acanthamoeba* spp. (Fiori et al. 2006). More recently, KP research has expanded into formulation-based antiparasitic applications. In a recent 2025 study, KP-loaded hyaluronate/chitosan nanoparticles reduced *Toxoplasma gondii* infection parameters *in vitro* and appeared to enhance antiparasitic activity relative to free KP against intracellular parasites, highlighting formulation strategies as a potential route to enhance activity against intracellular pathogens, although partial activity was also observed with blank carrier nanoparticles (Bucella et al. 2025).

As shown in Table 1, antiparasitic activity is generally observed at higher micromolar concentrations than for fungal targets, further supporting the view that KP displays its strongest and most consistent potency against fungi. Nevertheless, there is also evidence that the monoclonal antibody from which KP was derived displayed broad microbicidal effects, including fungicidal, bactericidal, and protozoacidal activity, and KP appears to retain aspects of this wide-ranging efficacy (Magliani et al. 2008). Collectively, these findings indicate that KP can affect structurally diverse eukaryotic and prokaryotic pathogens, although the underlying molecular determinants remain incompletely understood.

Taken together, the published literature indicates that KP has been reported to exhibit activity across fungal, bacterial, viral, and protozoal systems (Fig. 3). However, Table 1 shows that both potency and depth of evidence vary substantially across these groups. Antifungal activity is supported by the most consistent low-concentration efficacy and the most extensive mechanistic and *in vivo* data and therefore represents the most credible translational direction. By contrast, antibacterial, antiviral, and antiparasitic activities are more variable, often require higher concentrations, and remain comparatively less validated, indicating that these applications should currently be considered exploratory. Multi-kingdom antimicrobial activity is rarely observed in short peptides, making KP an unusual example of a peptide reported to affect fungi, bacteria, viruses, and protozoa. This broad efficacy may reflect either partially conserved target-recognition features or, more likely, multiple modes of action across different pathogen classes (Magliani et al. 2011).

## Mechanisms of action

**Figure 4:**
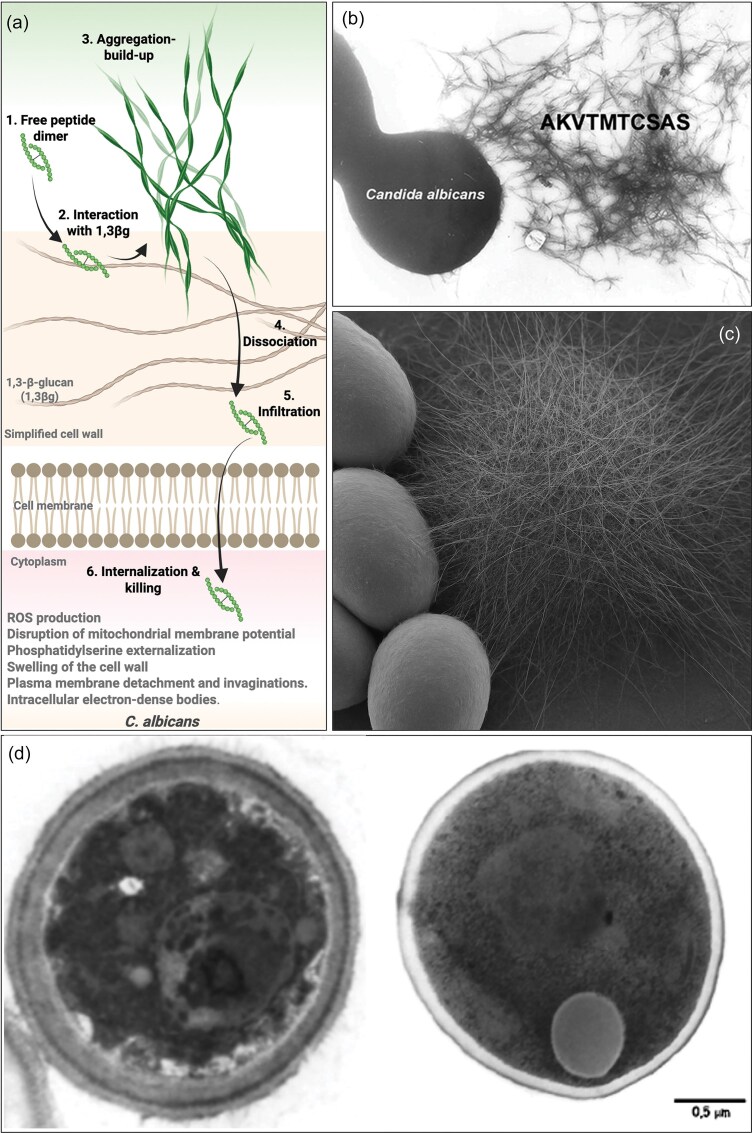
KP interaction with *C. albicans*. (a) Model of stepwise KP mechanism of action on *C. albicans;* (b) Electron micrograph showing KP fibrillar assemblies surrounding *C. albicans* cells, extracted from Pertinhez et al. (2009); (c) Schematic illustration of KP fibrillar assemblies adjacent to *C. albicans* cells and interacting with the cell surfaces. (d) Transmission electron microscopy of *C. albicans* following treatment with KP (left) or scrambled peptide control (right). KP-treated cells display pronounced structural alterations, including cell wall swelling with an electron-dense middle layer, plasma membrane collapse, chromatin condensation, and nuclear fragmentation., from Rodrigues et al. (2009) .

**Figure 5:**
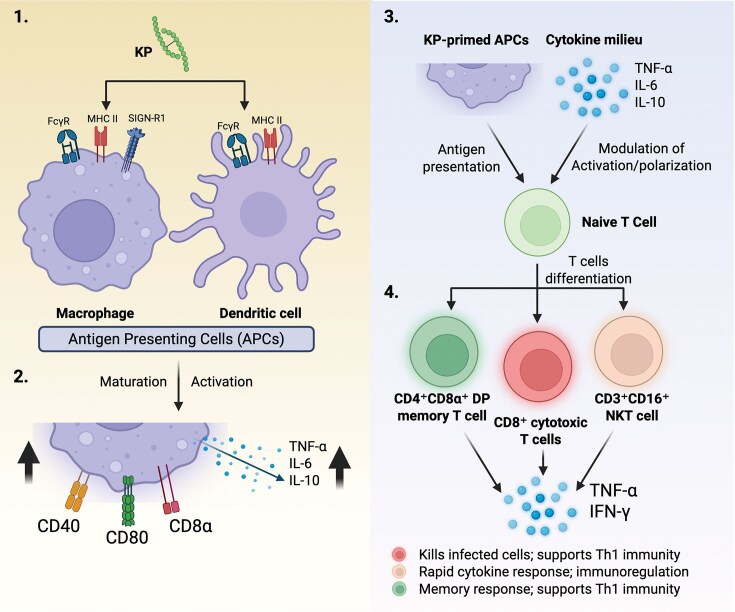
KP-mediated activation of innate and adaptive immune responses. KP interacts with dendritic cells and macrophages through receptors such as SIGN-R1, MHC II, and Fcγ (CD16/32), promoting antigen-presenting cell maturation and secretion of pro-inflammatory cytokines including TNF-α and IFN-γ. These signals drive the activation of CD3⁺CD16⁺ natural killer T cells, CD8α⁺β⁺ cytotoxic T cells, and CD4⁺CD8α⁺ double-positive T cells, fostering a Th1-type immune profile. Together, these effects indicate that KP functions not only as a direct antimicrobial agent but also as an immunomodulatory peptide capable of modulating host defense responses.

KP exerts antimicrobial activity through a combination of direct effects on microbial cells and modulation of host immune responses. Current evidence supports a multistep mechanism involving surface recognition, cellular uptake, intracellular stress induction, reversible self-assembly (Fig. 4), and immunomodulatory activity (Fig. 5).

### Surface recognition and cellular entry

KP activity begins with interaction with microbial surfaces. In fungi, KP recognizes β-1,3-glucans, major structural polysaccharides of the fungal cell wall (Polonelli et al. 2003). Competitive inhibition by the soluble β-glucan laminarin further supports glucans as a primary binding target (Ciociola et al. 2021b). Evidence also indicates that the disulfide-linked dimeric form represents the biologically active species required for efficient binding and fungicidal activity (Magliani et al. 2011).

Following binding, KP enters *Candida* cells and induces oxidative stress responses. Propidium iodide uptake assays and confocal microscopy studies showed that KP enters viable cells and induces killing more slowly than classical pore-forming peptides that directly disrupt membrane integrity (Paulone et al. 2017, Ciociola et al. 2021b). In bacteria, which lack β-1,3-glucans, KP may instead interact with alternative surface glycans or membrane-associated structures, although the precise receptors remain unknown. One hypothesized target is cyclic β-1,2-glucans present in the periplasm of many Proteobacteria (Guidolin et al. 2018). Alternatively, KP may employ partially glucan-independent mechanisms involving interactions with other cell envelope components, suggesting that uptake pathways may differ depending on the target organism. In summary, KP first localizes to microbial surfaces via glucan binding, followed by envelope destabilization that facilitates KP internalization and further action.

### Intracellular targeting and induced cell death

Once internalized, KP induces intracellular stress responses that lead to loss of viability. In *Candida*, KP treatment is associated with reactive oxygen species accumulation and rapid loss of mitochondrial membrane potential (Magliani et al. 2008, Paulone et al. 2017, Ciociola et al. 2021b). These effects are consistent with apoptosis-like programmed cell death pathways described in fungi. Additional markers, including phosphatidylserine externalization, further support activation of regulated cell death rather than nonspecific lysis (Ciociola et al. 2021b). However, KP-mediated killing may not depend exclusively on apoptosis. Certain analogues, such as K10S, retain strong fungicidal activity while inducing weaker apoptotic signatures, suggesting that multiple lethal mechanisms contribute to activity.

In protozoa such as *Leishmania* spp., KP inhibits growth without evidence of classical membrane lysis or DNA fragmentation. Ultrastructural studies have reported vacuolization and cytoplasmic degradation, findings more consistent with autophagic or non-apoptotic death pathways (Scutera et al. 2005, Savoia et al. 2006). These data support the view that KP can function as an intracellularly active antimicrobial peptide capable of triggering different stress-associated death programs depending on the target organism.

### Self-assembly and localized auto-delivery

A distinctive feature of KP is its ability to undergo reversible self-assembly into fibrillar supramolecular structures. Although this process can occur spontaneously, it is strongly accelerated by interaction with β-1,3-glucans such as laminarin or fungal cell-wall glucans (Pertinhez et al. 2009, Magliani et al. 2011). Current evidence suggests that KP exists in a dynamic equilibrium between monomeric peptide, disulfide-linked dimers, oligomeric intermediates, and higher-order fibrillar assemblies (Fig. 2d). While the structures of these intermediates remain incompletely characterized, this behaviour is consistent with the reversible supramolecular organization observed experimentally (Pertinhez et al. 2009).

Upon glucan binding, KP dimers assemble into fibrils stabilized by intermolecular hydrogen bonding, forming a localized peptide network at the microbial surface. These fibrillar assemblies have been proposed to function as temporary reservoirs that gradually release active peptide species. Such an auto-delivery mechanism may protect the peptide from proteolysis, increase local retention, and prolong antimicrobial activity. Electron microscopy studies showing fibrillar material associated with treated fungal cells further support this model (Pertinhez et al. 2009).

Although this mechanism still requires validation under physiological conditions, relatively few antimicrobial peptides have been reported to combine direct antimicrobial activity with target-triggered self-assembly. This property may provide a useful framework for the development of peptide-based delivery systems.

### Immunomodulatory effects

Beyond its direct antimicrobial activity, KP has also been reported to modulate host immune responses, indicating that KP may influence host defense pathways in addition to direct antimicrobial activity (Fig. 5). Available evidence indicates that KP interacts with innate immune cells, including dendritic cells and macrophages, through interactions involving SIGN-R1-reactive molecules, MHC class II, and Fcγ receptors (CD16/32) (Cenci et al. 2006). In murine antigen-presenting cells, these interactions were associated with phenotypic maturation, including increased expression of CD40 and CD80, along with enrichment of CD8α+ dendritic cell phenotypes (Cenci et al. 2006).

Functional studies further suggest that KP promotes pro-inflammatory and Th1-associated immune responses. Exposure of antigen-presenting cells to KP induced cytokine production, including TNF-α, consistent with activation of cellular immune pathways (Cenci et al. 2006). In a swine model, intramuscular administration of KP increased circulating CD3⁺CD16⁺ natural killer T cells and CD8αβ⁺ cytotoxic T-cell populations, together with dose-dependent increases in IFN-γ and TNF-α levels (Ferrari et al. 2020). ELISPOT analyses additionally showed enhanced IFN-γ responses to viral antigens, suggesting that KP may enhance antigen-specific cellular immunity.

KP treatment was also associated with increased frequencies of CD4⁺CD8α⁺ double-positive T cells in pigs, a population linked to memory and effector functions in this species (Ferrari et al. 2016, 2020). These studies suggest that KP can influence both microbial viability and host immune responses.

Although the available evidence remains limited and is derived from a relatively small number of studies, these observations support further investigation of KP and related derivatives as potential adjunctive immunotherapeutic agents, particularly in infections where host immune responses contribute substantially to microbial clearance. Similar dual antimicrobial and immunomodulatory properties have also been reported for several host-defense peptides.

## Derivatives and analogues of KP

Multiple KP derivatives have been generated to define structure-activity relationships and improve potency, kinetics, or stability. As summarized in Table 2, even minimal sequence modifications can substantially alter potency, kinetics, and target specificity, demonstrating the feasibility of rational design from KP scaffold. These modifications include amino acid substitutions, sequence deletions, stereochemical inversion, and rational in silico design.

**Table 2:** Representative KP derivatives and engineered analogues: sequence modifications, functional outcomes, and translational relevance.

Variant	Modification/design strategy	Key functional outcome	Mechanistic insight	Translational relevance	Reference
KP (native scaffold)	Decapeptide AKVTMTCSAS; activity strongly associated with disulfide-linked dimerization, while reduced/monomeric forms show diminished potency	Broad antifungal activity; self-assembly; immunomodulatory effects	Dimerization appears central to target binding, supramolecular assembly, and optimal bioactivity	Establishes native KP as the foundational scaffold for engineering; highlights importance of redox state and formulation control	Polonelli et al. 2003, Pertinhez et al. 2009, Magliani et al. 2011
K10S	N-terminal Ala → Lys substitution (increased cationicity)	Improved antifungal potency; faster killing in some assays	Charge enhancement improves microbial surface engagement	Lead optimization candidate for fungal indications	Ciociola et al. 2021b
H10S	N-terminal Ala → His substitution	Enhanced or retained antifungal activity; preserved immunological effects	Histidine may support pH-responsive charge behavior and uptake	Potentially useful in acidic infection niches	Ciociola et al. 2021b
Scrambled KP controls	Same amino acids, randomized order	Inactive or markedly reduced activity	Activity is sequence-dependent, not nonspecific hydrophobicity	Supports specific mechanism rather than detergent-like lysis	Polonelli et al. 2003
D-KP/all-D enantiomer	Full D-amino acid inversion	Reduced activity in some systems	Suggests stereospecific interaction with biological targets	Stability gains alone may not preserve efficacy	Travassos et al. 2004, Magliani et al. 2011
K10T-TT and K10S-SS	In silico optimized derivatives	Retained or improved antifungal activity; activity against biofilm-producing cephalosporin-resistant *E. coli*	Demonstrate that the KP scaffold tolerates computational redesign and can extend beyond fungi with sequence optimization	Candidate analogues for resistant infections, biofilm-associated disease, and expanded pathogen targeting	Ciociola et al. 2021a, Artesani et al. 2024
SP6	Related antibody-derived peptide from independent system	Antifungal activity with KP-like properties	Suggests convergent emergence of compact anti-infective motifs	Expands concept beyond single KP lineage	Kabir et al. 2011
Plant-expressed KP constructs	Recombinant expression in *Nicotiana benthamiana*	Feasible heterologous production of active peptide	Demonstrates manufacturability routes	Supports scalable biologics/agri-biotech production concepts	Donini et al. 2005

### N-terminal modifications

The native KP starts with an N-terminal alanine (A1). To investigate the role of the N-terminal residue, Ciociola et al. systematically replaced alanine with different amino acids (Ciociola et al. 2021b). Substitution with the basic residues histidine (H10S) or lysine (K10S) resulted in approximately twofold lower EC_50_ values against *C. albicans* SC5314 compared to KP. In contrast, substitution with hydrophobic leucine (L10S) markedly reduced activity, whereas serine (S10S) and tyrosine (Y10S) caused only minor reductions. Proline substitution (P10S) largely retained wild-type activity. These findings suggest that a positively charged N-terminus may promote candidacidal activity, potentially by increasing electrostatic interactions with negatively charged fungal cell surfaces. Consistent with this interpretation, K10S and H10S showed improved adhesion to yeast cells and faster initiation of killing. However, despite its high potency, H10S displayed slower killing kinetics than K10S. This difference affected *in vivo* efficacy in a *Galleria mellonella* candidiasis model, where K10S improved survival more effectively than H10S. Another analogue, K9S, lacking the N-terminal alanine, showed slightly reduced activity, suggesting that the presence of the N-terminal residue contributes to optimal function. Overall, these results demonstrate that N-terminal charge and residue identity influence both antimicrobial potency and killing kinetics.

### *In silico*-designed peptides (ISDPs)

In addition to single-residue analogues, four K10S derivatives—R10S-RR, K10S-I, K10T-TT, and K10S-SS—were rationally designed to improve antimicrobial activity and structural stability through *in silico* modelling (Ciociola et al. 2021a). Among these, K10T-TT and K10S-SS contain multiple substitutions, including additional charged residues, and adopt defined secondary structures: a β-sheet for K10T-TT and an α-helix for K10S-SS. Both peptides were initially developed for antifungal applications and were later evaluated against ESBL-producing *E. coli*. In planktonic killing assays, both peptides showed submicromolar EC50 values. In biofilm inhibition assays on polystyrene, EC_50_ values were in the micromolar range. Moreover, K10T-TT reduced biomass and viable cells in mature biofilms formed on stainless steel, as confirmed by 3D CLSM and scanning electron microscopy (Artesani et al. 2024).

### Self-assembly differences

The N-terminal variants also differed in self-assembly and target interactions. Circular dichroism analysis showed that some analogues, including H10S, L10S, and Y10S, displayed CD spectral features consistent with increased β-sheet organization, indicating a greater tendency to form ordered structures in solution. In contrast, KP and several other variants remained largely disordered in solution until interacting with glucan or target cells. Laminarin neutralization assays further revealed differences in glucan dependence. H10S and K10S were fully neutralized at lower laminarin concentrations than KP, whereas S10S retained partial activity even at the highest concentration tested (Ciociola et al. 2021b). These findings suggest that most variants rely on glucan binding for activity, while some may involve additional interactions. In terms of self-assembly, all active analogues still require dimerization via the cysteine to function; replacing or removing the cysteine abrogates candidacidal activity (this was shown also for other antibody-derived peptides in earlier alanine-scanning work where Cys replaced by Ala led to loss of function) (Magliani et al. 2015). Thus, the disulfide-linked dimer is a fundamental active unit for KP and its analogues.

### D-amino acid analogue

To investigate the role of chirality and protease resistance, a D-enantiomer of KP containing only D-amino acids was synthesized. Surprisingly, the D-peptide showed approximately eight-fold lower antifungal activity than the native L-form. In *P. brasiliensis*, substantially higher amounts of D-KP were required to achieve fungicidal effects (Travassos et al. 2004). These findings indicate that the mechanism of KP is at least partly stereospecific, potentially involving interactions with chiral targets such as β-glucan structures or cellular receptors. Although D-peptides are generally more resistant to proteolysis, the altered chirality may impair KP self-assembly or target binding. Thus, conversion to an all-D peptide does not improve KP activity.

### Scrambled and other control peptides

A scrambled-sequence peptide (SP: MSTAVSKCAT), containing the same amino acids as KP in a different order, is commonly used as a negative control (Polonelli et al. 2003, Travassos et al. 2004, Fiori et al. 2006, Paulone et al. 2017). SP consistently lacks antimicrobial activity, demonstrating that KP function depends on its specific sequence rather than amino acid composition alone. This finding further supports the idea that KP acts through specific molecular interactions rather than nonspecific toxicity. In addition to KP, several other antibody-derived peptides with antimicrobial or immunomodulatory activity have been identified (Magliani et al. 2011, 2015, Ciociola et al. 2016). For example, the undecamer peptide T11F, derived from a human IgM constant region, exhibits candidacidal activity without toxicity toward mammalian cells (Magliani et al. 2015). Similarly, peptides such as VH CDR3 and N10K, derived from immunoglobulin regions, display immunomodulatory properties similar to those of KP (Gabrielli et al. 2009, 2012). Although these peptides are not direct KP analogues, they belong to the broader class of antibody-derived antimicrobial peptides and illustrate that immunoglobulin sequences can carry bioactive cryptides with antimicrobial functions.

### Convergent analogues

In addition to KP derivatives, other antibody-derived peptides with sequences similar to KP have been identified. Kabir et al. (2011) described SP6, a decapeptide (AKVTITCSVS) derived from the complementarity-determining region of a single-chain variable fragment anti-idiotypic antibody that mimics the HM-1 killer toxin from *Cyberlindnera mrakii* (Kabir et al. 2011). Like the parental toxin of KP, HM-1 recognizes β-1,3-glucans as a primary receptor. SP6 differs from KP by two amino acid substitutions but retains the cysteine residue required for disulfide-linked dimerization. Despite these sequence differences, SP6 displays similar biological activity, particularly against *Candida* and *Cryptococcus* species, with micromolar IC_50_ values. Its activity is also neutralized by laminarin, indicating a conserved glucan-dependent mechanism. The similarities between KP and SP6 suggest the existence of a shared functional motif likely driven by the shared β-1,3-glucan receptor specificity of their parental toxins.

### Expression fusions

Beyond point substitutions, researchers have also explored fusing or displaying KP on carriers. One approach was using a plant virus (Potato virus X) as a vector to display KP on its coat protein in plants (Donini et al. 2005). The resulting chimeric virus particles (CVPs) had multiple copies of KP on their surface. These CVPs showed enhanced antimicrobial activity relative to free KP in assays against phytopathogens.

Overall, derivative studies indicate that KP is a tractable scaffold for peptide engineering that can potentially be adapted from a primarily antifungal scaffold toward broader anti-infective applications. Future optimization will likely depend less on maximizing intrinsic potency alone and more on balancing activity, kinetics, stability, selectivity, and formulation compatibility.

## Clinical potential and applications

KP exhibits several properties that support further investigation as an anti-infective peptide, particularly in fungal infections. Although it is not in clinical use, preclinical studies have demonstrated activity in both systemic and topical infection models.

### *In vivo* efficacy

KP has demonstrated therapeutic activity in several experimental fungal infection models, although the available evidence remains limited to relatively small preclinical studies. The strongest *in vivo* data currently support antifungal applications, particularly in candidiasis and other mucosal or localized fungal infections.

In an early study, Polonelli et al. (2003) evaluated KP in both mucosal and systemic candidiasis models. In estrogen-treated rats with vaginal *C. albicans* infection, intravaginal administration of KP accelerated fungal clearance and reduced vaginal fungal burden compared with untreated or scrambled-peptide controls. Therapeutic effects were also observed against a fluconazole-resistant *C. albicans* strain. In systemic candidiasis models, KP treatment significantly prolonged survival in both immunocompetent BALB/c and SCID mice challenged intravenously with lethal *C. albicans* inocula, with most KP-treated animals surviving beyond 60 days whereas control animals died within the first week. KP activity in these models was broadly comparable to fluconazole under the experimental conditions tested (Polonelli et al. 2003). Travassos et al. (2004) subsequently demonstrated efficacy in experimental paracoccidioidomycosis. In a murine pulmonary infection model caused by *Paracoccidioides brasiliensis*, KP treatment reduced fungal burden in the lungs, liver, and spleen. The study also showed that an all-D enantiomer of KP retained antifungal activity but required substantially higher concentrations to achieve comparable effects, supporting at least partial stereospecificity of KP-mediated fungal killing.

Localized therapeutic activity was further explored by Cafarchia et al. (2014) in dogs with naturally occurring *Malassezia pachydermatis* otitis externa. Daily topical administration of KP ear drops for eight days reduced fungal burden and improved clinical symptoms, whereas scrambled-peptide and untreated control groups showed little improvement. No relapse was reported during the two-month follow-up period, although the study involved a limited number of animals and larger studies will be required to confirm efficacy and reproducibility.

These studies indicate that KP can exert therapeutic antifungal activity *in vivo* across multiple infection settings. However, the current evidence base remains limited by small cohort sizes, short treatment windows, and the absence of pharmacokinetic or long-term toxicology studies. At present, topical or localized antifungal applications appear more immediately plausible than systemic therapeutic development.

KP also inhibited *C. albicans* biofilms on abiotic surfaces, including silicone catheters (Paulone et al. 2017), supporting potential applications in the prevention of device-associated fungal infections.

### Safety and toxicity

Across available *in vitro* and *in vivo* studies, KP has shown low apparent toxicity toward mammalian cells. The peptide did not induce significant haemolysis or markedly affect mammalian cell viability at concentrations exceeding those required for antifungal activity (Magliani et al. 2011). KP also exhibited limited toxicity in antiviral assays, with high selectivity indices reported in HSV models, particularly against HSV-2 (Sala et al. 2024). Although KP can promote pro-inflammatory immune responses, including TNF-α and IFN-γ production, available animal studies have not reported overt inflammatory toxicity or obvious treatment-related adverse effects (Polonelli et al. 2003, Travassos et al. 2004, Cenci et al. 2006, Cafarchia et al. 2014, Ferrari et al. 2016, 2020). Nevertheless, comprehensive toxicological, pharmacokinetic, and biodistribution studies remain necessary before clinical translation.

### Formulation strategies

KP is a small peptide that can be readily synthesized by solid-phase methods and formulated in aqueous solutions or lyophilized preparations. Its self-assembly upon interaction with β-glucans may support localized retention and sustained activity at fungal infection sites (Pertinhez et al. 2009, Magliani et al. 2011, Ciociola et al. 2021b).

Localized delivery approaches, including creams, hydrogels, catheter coatings, or mucosal formulations, currently appear more feasible than systemic administration. More complex delivery systems, including virus-based display platforms and nanoparticle formulations, have also been explored experimentally (Donini et al. 2005, Bucella et al. 2025), although their translational practicality remains uncertain. In particular, hyaluronate/chitosan nanoparticles were recently reported to enhance KP activity against intracellular *Toxoplasma gondii* compared with the free peptide, supporting further investigation of carrier-based approaches for intracellular delivery applications (Bucella et al. 2025).

### Resistance potential

KP targets conserved fungal cell-wall components and induces intracellular stress responses, which may reduce the likelihood of rapid resistance development. *Candida* strains resistant to fluconazole or amphotericin B remained susceptible to KP (Polonelli et al. 2003, Paulone et al. 2017), and no resistance was observed following repeated HSV exposure to subinhibitory peptide concentrations (Sala et al. 2024). In addition, a genome-wide *Saccharomyces cerevisiae* deletion library failed to identify mutants with reduced KP susceptibility (Conti et al. 2008b).

However, these observations remain limited, and long-term resistance evolution studies have not yet been performed. Similarly, although KP analogues showed activity against ESBL-producing *E. coli* and biofilms (Artesani et al. 2024), the antibacterial dataset remains comparatively small relative to the antifungal literature.

## Challenges and development considerations

Several challenges remain before KP can be considered for clinical development. Like many small peptides, KP would be expected to undergo rapid proteolytic degradation and renal clearance, which may limit systemic exposure. Although self-assembly at glucan-rich sites could improve local retention (Pertinhez et al. 2009), this mechanism may not protect the peptide during circulation. Strategies such as backbone modification, PEGylation, or optimized delivery systems may improve peptide stability and pharmacokinetic behaviour.

Delivery and bioavailability also remain important limitations. Localized applications are currently more plausible than systemic administration, particularly for fungal skin, mucosal, or device-associated infections. Delivery platforms will likely need to preserve or support formation of the biologically active dimeric state of KP.

**Figure 6:**
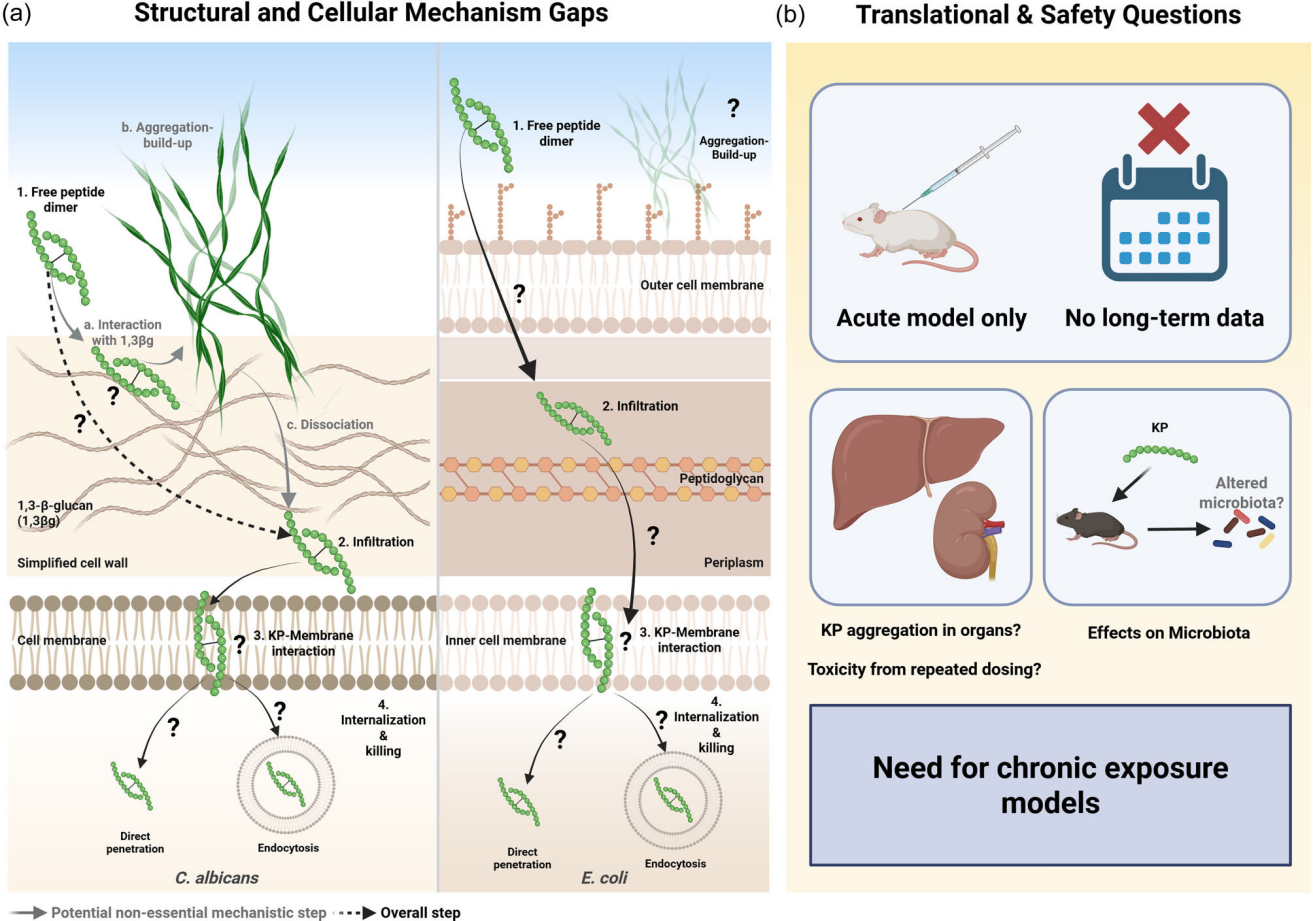
Open questions and knowledge gaps in Killer Peptide research. (a) Structural and cellular mechanism gaps: (i) the structural basis of KP-β-glucan interactions and the molecular architecture of fibrillar assemblies; (ii) the relative contribution of soluble dimers versus fibrillar aggregates to antimicrobial activity *in vivo*; (iii) the mechanism of KP transit through the fungal cell wall toward the plasma membrane; (iv) KP-membrane interactions and mechanisms of cellular internalization; and (v) the occurrence and functional relevance of peptide aggregation in non-fungal systems lacking β-1,3-glucans. (b) Pharmacokinetic, immunogenicity, and microbiome-related questions associated with repeated or systemic administration.

Although KP has shown low toxicity in available studies, additional evaluation of repeated dosing, immunological effects, and tissue distribution is still required. Likewise, the impact of prolonged exposure on host-associated microbiota composition and function has not been examined. Several mechanistic and translational questions also remain unresolved (Fig. 6). The structural basis of KP interaction with β-glucans and downstream cellular targets is incompletely defined, and the pathways governing cellular uptake may differ among fungi, bacteria, viruses, and protozoa. It also remains unclear whether fibrillar self-assembly is required for activity or primarily enhances peptide retention and stability at infection sites.

Overall, current evidence supports KP primarily as a promising antifungal and immunomodulatory scaffold rather than a broadly validated multi-kingdom therapeutic platform. Future progress will depend on improved mechanistic understanding, optimized formulations, and rigorous *in vivo* validation across clinically relevant models.

## Concluding remarks

KP represents an unusual example of an antibody-derived antimicrobial peptide whose activity extends beyond the conventional framework of membrane-disruptive AMPs. Rather than acting through a single dominant mechanism, current evidence suggests that KP combines target recognition, intracellular stress induction, reversible self-assembly, and host immunomodulatory effects within a minimal molecular scaffold. This mechanistic complexity places KP at the intersection of antimicrobial peptides, host-defense peptides, and supramolecular peptide systems.

Importantly, KP also illustrates an alternative route to anti-infective peptide discovery. Unlike many antimicrobial peptides identified through innate immunity or genome-mining approaches, KP emerged through anti-idiotypic mimicry of a fungal killer toxin, highlighting how functional peptide scaffolds can arise from antibody engineering strategies. More broadly, the anti-idiotypic vaccination and antibody-mimicry approach that led to KP may represent a versatile platform for uncovering bioactive cryptides with diverse biological functions extending beyond anti-infective activity. Similar strategies could potentially be exploited to generate peptides with immunomodulatory, anticancer, receptor-targeting, or other therapeutic properties derived from functional antibody repertoires.

The ability of relatively small sequence modifications to alter potency, kinetics, glucan dependence, and self-assembly further emphasizes the tractability of the KP scaffold for rational peptide design. At present, the strongest evidence supports KP primarily as an antifungal and immunomodulatory scaffold, particularly for localized or biofilm-associated applications. However, many mechanistic questions remain unresolved, including the relationship between peptide assembly, cellular uptake, and intracellular targeting. Future studies integrating structural biology, peptide engineering, and rigorous infection models will determine whether these distinctive properties can be translated into clinically useful therapeutic systems.
